# Kidney-Sparing Approaches in Upper Tract Urothelial Cancer: A Narrative Review

**DOI:** 10.7759/cureus.103303

**Published:** 2026-02-09

**Authors:** Charalampos Kefalas, Nikolaos Datseris, Konstantinos Sitaridis, Evangelos Mourtzilas, Apostolos Papalakis

**Affiliations:** 1 Urology Department, General Hospital of Thessaloniki 'O Agios Dimitrios', Thessaloniki, GRC

**Keywords:** endoscopic ablation, kidney-sparing surgery, nephron-sparing management, nephroureterectomy, photodynamic therapy, renal function preservation, risk stratification, ugn-101, upper tract urothelial carcinoma, ureterectomy

## Abstract

Upper tract urothelial carcinoma (UTUC) is a relatively rare neoplasm. Historically, radical nephroureterectomy (RNU) has been the gold-standard treatment; however, its association with postoperative renal dysfunction and reduced quality of life has driven a paradigm shift toward kidney-sparing surgery (KSS). This literature review synthesises late evidence regarding nephron-sparing techniques for UTUC, such as endoscopic ablation, segmental and total ureterectomy and novel minimal invasive modalities. Clinical, radiologic, and molecular parameters are examined in order to refine risk stratification, which is crucial for selecting appropriate patients who may benefit from KSS. Even though endoscopic ablation is associated with higher local recurrence rates, it demonstrates comparable cancer-specific and overall survival to RNU in low-risk UTUC and selected high-risk patients. Segmental and total ureterectomy, if technically feasible, accomplish similar oncologic outcomes with RNU and are superior regarding renal function preservation. Novel kidney-sparing therapies such as intraluminal chemoablation with UGN-101 and vascular-targeted photodynamic therapy show encouraging efficacy and safety in early trials. Perioperative chemotherapy and immunotherapy may expand the indication for kidney-sparing approaches in the near future, combined with emerging biomarker-driven models such as the aristolochic acid (AA) mutational signature. Despite these advances, long-term oncologic outcomes, optimal adjuvant regimens, and molecularly guided treatment algorithms remain incompletely defined. In order to verify oncologic efficacy, systematise follow-up protocols, and establish personalized therapeutic pathways guided by molecular patterns, future multicenter prospective studies are essential.

## Introduction and background

Epidemiology

Upper tract urothelial cancer (UTUC) is a relatively uncommon neoplasm, accounting only 5-10% of urothelial carcinomas (UC) and 5-7% of all renal neoplasms [1]. Due to its low incidence, which has been estimated at 1-2 cases per 100,000, UTUC is underrepresented in large randomised control trials (RCTs) and, as a result, guidelines for its management leverage heavily on the management of lower tract urothelial cancer (LTUC) [2,3].

Even though morphologically similar, UTUC and LTUC seem to have genetic and epigenetic differences that make them different disease entities requiring distinct management [4]. Ureteral and bladder urothelial cells diverge embryologically, histologically and molecularly as they originate from different embryological tissues (mesoderm and endoderm, respectively); they have discrete uroplakin content, keratin expression pattern, propensity to keratinase, and UTUCs seem to present more microsatellite instability and hypermethylation [4,5]. Moreover, UTUC presents with muscle invasion in more than 60% of patients at the time of diagnosis, whereas only 15-25% of LTUC present with invasive disease, significantly affecting the prognosis of the disease [6].

Considering these findings, the European Association of Urology (EAU) has developed exclusive guidelines for UTUC [7]. Based on a precise risk stratification plan, patients characterized as low-risk and even carefully selected high-risk patients seem to benefit from nephron-sparing approaches [8]. Low-risk disease is defined by tumor size <2 cm, negative high-grade cytology, low-grade ureteroscopy (URS) biopsy, unifocal disease and no invasive aspect on CT [7].

Rationale for kidney-sparing approaches

Historically, radical nephroureterectomy (RNU), a procedure involving the complete removal of the affected kidney, ureter, and a cuff of the bladder, has been the gold standard treatment for UTUC. However, RNU imposes several risks as it is associated with impaired renal function outcomes, deterioration in quality of life and may lead patients with preexisting renal conditions, solitary kidneys or bilateral UTUC to kidney failure requiring dialysis [9]. The aforementioned increase surgery-related morbidity in patients undergoing RNU, while the glomerular filtration rate (GFR) in 55% of these patients will decrease in the long-term [10]. Moreover, renal insufficiency limits eligibility for systemic therapies that can be offered, whether in adjuvant or salvage settings [8].

Kidney-sparing surgery (KSS) has demonstrated comparable oncologic outcomes when compared to RNU [11]. On the contrary, KSS has been shown to maintain better GFR, reducing the progression to chronic kidney disease (CKD) and complications related to renal insufficiency, especially in patients with preexisting comorbidities [12]. Furthermore, the preservation of renal function related to KSS seems to lead to a lower risk of cardiovascular disease development and may preclude cardiovascular morbidity and fatal events, especially in the elderly population [13].

Considering the aforementioned, there has been a paradigm shift towards nephron-sparing approaches in low-risk UTUC and in carefully selected high-risk patients, such as those with solitary kidney, low preoperative GFR, increased cardiovascular risk and frail patients [1,3,7,12]. Emerging retrospective series and prospective data suggest that kidney-sparing approaches, including endoscopy and segmental ureterectomy, may achieve oncologic outcomes comparable to RNU while preserving renal function, in selected patients [13].

Scope and methods of the review

This literature review aims to critically evaluate the spectrum of kidney-sparing techniques currently applied in UTUC, encompassing endoscopic management, segmental and total ureterectomy, and emerging chemoablative and photodynamic therapies. A PubMed literature review was conducted in English-language research papers using the following search terms: [(kidney-sparing OR nephron sparing) and (management or therapy or therapeutic interventions) and (upper tract urothelial carcinoma or UTUC)]. Papers published in the past 10 years were primarily included in our search. Subsequently, a snowball search was performed in areas where there was not enough data from our initial research. Whenever available, systematic reviews and meta-analyses were prioritized, along with randomized controlled trials evaluating new treatments. The study selection and synthesis were qualitative. This review consolidates and synthesizes evidence on oncologic safety, functional outcomes, and patient-centered factors influencing treatment success. Further, it examines clinical and molecular criteria for patient selection and explores adjunct therapies improving outcomes post kidney-sparing interventions. Moreover, it addresses systemic and topical adjuvant therapies used post kidney-sparing surgery to control residual or recurrent disease. Finally, it outlines the importance of robust risk stratification tools and personalized treatment algorithms to optimize patient selection and maximize therapeutic efficacy.

## Review

Risk stratification and patient selection

Clinical and Imaging Criteria for Kidney-Sparing Suitability

Hematuria, either macroscopic or microscopic, represents the predominant clinical manifestation of upper tract urothelial carcinoma, documented in approximately 70% to 80% of patients at initial presentation [14]. Flank pain constitutes a less frequent presenting symptom, occurring in 20% to 40% of affected individuals [15]. The clinical presentation with a palpable abdominal or lumbar mass is comparatively rare, observed in more advanced disease stages [1]. Both flank pain and palpable masses are recognized as clinical indicators that frequently correlate with locally advanced disease and are associated with an unfavorable prognosis [14,16]. Additionally, recent multicenter data indicate that patients presenting with symptomatic hydronephrosis demonstrate significantly reduced overall survival and cancer-specific survival compared to those presenting with hematuria alone [17].

Accurate risk stratification is fundamental to select appropriate candidates for kidney-sparing management in UTUC. Patients with low-risk UTUC are considered suitable for kidney-sparing strategies, while high-risk patients are generally recommended for radical surgery [12]. According to the European Association of Urology (EAU) guidelines, low-risk disease is typically characterized by unifocal lesions, tumor size less than 2 cm, low-grade histology, and absence of invasive features on imaging or endoscopy. Conversely, high-grade disease, either by URS biopsy or cytology, radiologic evidence of invasion and histological subtypes strongly mark high-risk profiles where nephron-sparing approaches may compromise oncologic outcomes, while multifocal disease, tumor size bigger than 2 cm and hydronephrosis pose weak criteria for high-risk definition when a low-grade tumor is present [5].

Imaging techniques, particularly computed tomography urography (CTU), play a pivotal role in preoperative assessment and staging. CTU demonstrates high diagnostic accuracy (94.2%-99.6%) for UTUC, surpassing older techniques like intravenous urography, and is currently the first-line imaging modality in suspected cases [18]. Key imaging features such as tumor size, location, and invasion depth are evaluated to inform surgical planning. Complementing imaging, diagnostic ureteroscopy provides direct visualization and tissue sampling vital for confirming tumor grade and invasion status, facilitating tailored treatment plans. Ureteroscopy also helps prevent overtreatment by ruling out benign conditions mimicking UTUC [19].

Tumor grade, determined via biopsy, significantly influences risk stratification, with low-grade lesions frequently considered appropriate for conservative management as they tend to be non-muscle invasive at the time of the diagnosis. Tumor location affects surgical feasibility; distal ureteral tumors and those confined to the renal pelvis present varying opportunities and technical challenges for kidney preservation [20]. Tumor size remains a significant predictor of treatment outcomes; lesions exceeding 3 cm in diameter are linked with poorer prognosis and may contraindicate kidney-sparing surgery [21]. These features combined guide clinicians on the viability of nephron-sparing interventions.

Molecular and Biomarker-Based Risk Models

Molecular and systemic biomarkers represent rapidly emerging tools that may guide risk prediction and patient selection in UTUC henceforth. A distinctive aristolochic acid (AA) mutational signature marks a low-risk UTUC subtype, prevalent especially in certain East Asian populations that are exposed to AA-containing herbal medicines. This molecular signature is characterized by female predominance, multifocal disease patterns presenting due to field cancerization, and better cancer-specific survival rates, when compared to no-AA subtypes of UTUC [22]. Therefore, detection of this molecular signature via urinary cell-free DNA may offer a noninvasive diagnostic tool to guide the selection of low-risk patients suitable for nephron-sparing management and potentially immunotherapy regimens tailored to mutational profiles.

Systemic inflammatory biomarkers, namely neutrophil-to-lymphocyte ratio (NLR), platelet-to-lymphocyte ratio (PLR) and monocyte-to-lymphocyte ratio (MLR), have been proposed by Shao et al., and their preoperative elevated values seem to be associated with increased risks of mortality post RNU, even though their standalone prognostic value remains inconsistent. However, when combined with clinicopathological and molecular data, they may enhance risk stratification, guiding individualized management decisions [23].

Advanced optical imaging modalities, including optical coherence tomography (OCT) and confocal laser endomicroscopy (CLE), are emerging as powerful tools in tumor grading and staging. OCT offers minimally invasive, real-time assessment of urothelial layers, aiding in distinguishing tumor grades, which is critical in deciding kidney-sparing candidacy [24]. CLE enables in vivo histologic characterization during ureteroscopy, improving diagnostic accuracy and potentially enabling more precise endoscopic management [25]. These technologies may soon become standard adjuncts in UTUC diagnosis, fostering better patient selection for conservative treatments.

Development of Risk Stratification Models

A multicenter collaborative study published by Foerster et al. aims to refine risk stratification models using preoperative factors such as age, tumor grade, clinical T stage, hydronephrosis, tumor size and sessile tumor architecture to predict advanced pathological disease stages (≥pT2 or nodal involvement) [20]. Namely, invasion on imaging, biopsy cT1+ staging, endoscopic-based sessile tumor architecture and high-grade biopsy samples are strong predictors of muscle-invasive or lymph-node-positive disease. These patients should be excluded from nephron-sparing management using endoscopic techniques. These advances underscore the necessity of integrating clinical, radiologic, and endoscopic findings into robust predictive algorithms.

Accurate risk assessment minimizes undertreatment of aggressive disease and overtreatment of low-risk lesions, optimizing outcomes and preserving renal function when appropriate. Such stratification is critical to applying the emerging paradigm shift towards nephron-sparing management, ensuring oncologic safety without compromising quality of life [26].

Endoscopic management techniques

Ureteroscopic and Percutaneous Approaches

Endoscopic techniques include retrograde ureteroscopic and percutaneous interventions. URS laser ablation techniques have progressed considerably, especially with the thulium-holmium:YAG (TL-HL:YAG) duo laser which can be used in renal-conserving retrograde infrarenal surgery (RC-RIRS) to treat UTUC. Retrospective data show that laser ablation with URS TL-HL:YAG is both safe and effective when treating UTUC, with a kidney preservation rate of approximately 90% and predominantly mild complications, namely self-limiting hematuria [27]. Moreover, long-term follow-up showed that TL-HL:YAG duo laser was safe and oncologically noninferior to other combination laser energy technologies, but with better kidney preservation outcomes [27].

Other lasers can also be used, including thulium laser ablation. Thulium laser URS ablation, when compared to RNU, yields better postoperative renal function, reflected by lower creatinine levels postoperatively and shorter hospital stays. Despite higher recurrence rates, the approach remains acceptable with rigorous surveillance and endoscopic retreatments available for recurrences [28]. Moreover, when URS access is limited or the tumor is located in challenging anatomic areas, percutaneous resection techniques can be used [29].

Surveillance after endoscopic treatment is crucial due to the high local recurrence rates seen, with reported figures ranging from 28% to 85% [30]. Regular ureteroscopic examination and imaging are necessary, and salvage treatments are often feasible [30]. Despite the frequent need for repeated interventions, endoscopic management maintains renal function and can be a valuable option for selected patients.

Oncologic Outcomes of Endoscopic Management

Endoscopic nephron-sparing techniques of UTUC present comparable overall survival (OS) and cancer-specific survival (CSS) to RNU, in carefully selected low-grade patients, as shown by several studies (Table 1) [11, 13, 30, 31].

**Table 1:** E-KSS vs RNU E-KSS: endoscopic kidney-sparing surgery; RNU: radical nephroureterectomy; PFS: progression-free survival; IVRFS: intravesical recurrence-free survival; MFS: metastasis-free survival; DFS: disease-free survival; ESRD: end-stage renal disease; HR: hazard ratio; RR: risk ratio; OR: odds ratio; CI: confidence interval; EAU: European Association of Urology; UTUC: upper tract urothelial carcinoma; AJCC: American Joint Committee on Cancer; CSS: cancer-specific survival; eGFR: estimated glomerular filtration rate.

Study	Hendriks et al. (2022) [11]	Chen et al. (2021) [31]	Giulioni et al. (2024) [13]	Kawada et al. (2023) [30]
Design	Retrospective cohort; propensity-weighted (EAU risk strat.)	Retrospective cohort (registry-based)	Systematic review + meta-analysis (comparative studies)	Systematic review + meta-analysis (incl. adjusted HRs)
Setting / Country	Netherlands (tertiary center)	Taiwan	N/A (meta-analysis)	N/A (meta-analysis)
Population / Inclusion	Non-metastatic UTUC	Low-stage UTUC treated with RNU and all managed endoscopically	Localized UTUC (endoscopic KSS vs RNU)	UTUC (endoscopic KSS vs RNU)
N, total	186 renal units / 180 patients, 100 patients left after propensity score matching	356	2,284 patients (11 studies)	13 studies (adjusted HRs from 5 studies) N of patients included N/A
Groups	RNU 97; E-KSS 89 (RNU 50; KSS 50 propensity weighed cohort)	RNU 272; E-KSS 84	E-KSS 737; RNU 1,547	E-KSS vs RNU (N of patients included N/A)
Risk / staging notes	EAU risk used for weighting (exception KSS in high-risk after multidisciplinary meeting)	Staging: AJCC TNM; grading: WHO/ISUP	Retrospective comparative studies; selection bias	Primarily low-risk selection emphasized
Follow-up	Mean 42.33 mo (0–126)	Median 42 mo RNU; 33.5 mo E-KSS	N/A (varies by study)	N/A (varies by study)
Outcomes	PFS, MFS, CSS, OS; IVRFS; ipsilateral RFS; eGFR	OS, CSS, DFS, IVRFS, eGFR, risk of ESRD	5-yr OS/CSS/MFS, bladder recurrence rate	Adjusted HRs: OS/CSS/IVRFS
Key oncologic findings	Propensity-weighted (RNU vs KSS): PFS (4% vs 14%, log rank p = 0.147); MFS (28% vs 18%, log rank p = 0.217); OS (24% vs 24%, log rank p = 0.691); CSS (16% vs 14%, log rank p = 0.490)	Kaplan–Meier comparison: OS: similar (p=0.188), CSS: similar (p=0.493), DFS: inferior with endoscopic (p<0.001); Multivariable Cox (adjusted): RNU associated with better DFS: HR 0.078 (95% CI 0.018–0.336), p=0.001. Endoscopic approach did not affect OS, CSS in adjusted models	RNU vs KSS: OS (RR 1.14, 95% CI 0.97–1.33, p = 0.10), CSS (RR 1.13, 95% CI 0.81–1.58, p = 0.48); MFS (RR 0.63, 95% CI 0.33–1.20, p = 0.16);	Adjusted HRs (5 studies): OS HR 1.27 95% CI 0.75–2.16; CSS HR 1.37 95% CI 0.99–1.91
Recurrence findings	Propensity-weighted (RNU vs KSS): IVRFS (32% vs 52%, log rank p = 0.029); Ipsilateral RFS (8% vs 68%, log rank p = 0.000)	IVRFS similar (p=0.381)	Bladder recurrence rate (RR 0.96, 95% CI 0.78–1.18, p = 0.68)	Adjusted HRs (4 studies): IVRFS HR 0.98 95% CI 0.61–1.55
Renal function findings	eGFR difference largest at 3 mo (43.65 vs 54.53, p=0.000); no difference at 2 & 5 yr	Progression to ESRD (OR 0.745, 95% CI 0.422–1.315, p=0.31), eGFR decline smaller with E-KSS (8.24 vs 13.37, p=0.032)	N/A (not reported as pooled endpoint)	N/A (not pooled consistently)
Safety / burden	Procedures: mean 3.18 (RNU) vs 8.76 (KSS)	Hospital stay (median): 2 days (endoscopic) vs 8 days (RNU), p<0.001, Grade II Clavien-Dindo complications 4.76% vs 26.47% (E-KSS vs RNU) OR=7.445, p<0.001	N/A	N/A (not pooled consistently)
Limitations	Retrospective; follow-up burden differs by treatment	Retrospective, limited endoscopic cohort size	All retrospective; confounding/selection bias	Heterogeneous studies; selection bias

Meta-analyses reveal hazard ratios for OS and CSS approaching parity with RNU, reinforcing the viability of endoscopic treatment as a first-line approach in low-risk disease [30]. However, there seems to be a high local recurrence rate, and long-term surveillance is necessary in order to treat recurrences promptly [31].

The limitations of endoscopic techniques primarily relate to tumor multifocality and invasiveness. Multifocal tumors or high-grade lesions are associated with poorer outcomes and higher recurrence, making endoscopic management less suitable in such cases [32].

Adjuvant Endocavitary Therapies after Endoscopic Treatment

Adjuvant instillation therapies are used following kidney-sparing surgery to reduce tumor recurrence and progression. Mitomycin C local application after ureteroscopic tumor resection is feasible and well-tolerated with maintenance regimens improving recurrence-free survival. Instillations can be administered via percutaneous nephrostomy or cystoscopically placed ureteral catheters, demonstrating encouraging safety profiles [33]. Recent prospective trials indicate that single-dose upper tract instillation of mitomycin C immediately after therapeutic ureteroscopy reduces recurrence rates significantly compared to controls, with acceptable toxicity and complication rates [34].

Bacillus Calmette-Guerin (BCG) therapy, although effective in bladder carcinoma in situ, has shown limited efficacy in UTUC, and its role as adjuvant therapy in upper tract carcinoma remains not well defined. Maintenance regimens are also underexplored, posing challenges to routine clinical adoption [35]. Comprehensive meta-analyses conclude that adjuvant endocavitary instillation has not firmly demonstrated efficacy in reducing recurrence in non-muscle-invasive UTUC, with no differences observed between various regimens or instillation modalities. Ongoing development of novel agents promises to alter the therapeutic landscape in this domain [35].

Segmental and total ureterectomy

Surgical Techniques and Indications

Segmental ureterectomy (SU) and total ureterectomy (TU) represent kidney-sparing surgical techniques for ureteral tumors used when RNU is not feasible due to impaired renal function, solitary kidney or frail patients. SU targets the ureteral segment affected by the carcinoma, preserving the remainder of the urinary drainage and the ipsilateral kidney. TU, often combined with reconstructive techniques including ileal-ureteral substitution, is an option feasible when extensive ureteral involvement is present, namely in multifocal tumors or bilateral disease [36]. Long-term data indicate that ileal substitution is feasible and seems to preserve renal function with acceptable oncologic outcomes [37].

Patient selection for these management techniques is based primarily on tumor location, size and preoperative kidney function. Distal ureteral tumors are well-suited for distal ureterectomy with bladder cuff excision and ureteroneocystostomy, optionally combined with psoas hitch when needed, whereas in cases of an extensive ureteral defect, ureteral reimplantation using a Boari flap can be employed providing up to 10 cm of additional bladder mobilization [38,39]. When the tumor is located in the more proximal ureter, it is possible to perform SU and then reconstruct the ureter with an end-to-end uretero-ureterostomy, provided that both proximal and distal segments have been sufficiently mobilized to achieve a tension-free anastomosis [36]. More proximal or multifocal disease may require TU with reconstructive techniques [37].

Oncologic Efficacy and Safety Profile

Retrospective studies suggest that oncologic outcomes following SU and TU are comparable to those of RNU, with no significant differences observed in CSS or OS [36,37]. In a systematic review and meta-analysis conducted by Veccia et al., there were no statistically significant differences between the SU and RNU cohorts with respect to overall recurrence (P = .13), intravesical recurrence (P = .50), distant metastasis (P = .18), or cancer-specific mortality (P = .95). In the same direction, 5-year metastasis-free survival (MFS) and CSS did not differ significantly between the two surgical approaches, while in the subgroup of distal ureteric tumors, survival outcomes were comparable between segmental ureterectomy (SU) and radical nephroureterectomy (RNU). However, there was an advantage for RNU in terms of RFS (HR 1.26; 95% CI 1.07-1.49; P = .006), consistent with the 5-year RFS data, where SU was associated with a lower likelihood of remaining recurrence-free (OR 0.64; 95% CI 0.43-0.95; P = .03) [40]. In another systematic review and meta-analysis of the latest studies SU and RNU showed no significant differences in OS, RFS or MFS at 3 and 5 years (3-year OS HR 1.43 [0.09-22.35], 5-year OS HR 0.99 [0.94-1.04], 5-year RFS HR 1.02 [0.74-1.40], 5-year MFS HR 0.79 [0.41-1.50]), whereas SU consistently provided superior renal functional outcomes, with higher 1-year postoperative eGFR and better eGFR preservation at 1 month, 3 months and 1 year [41]. Furthermore, a study conducted by Fang et al. illustrated similar results as SU and RNU demonstrated comparable oncologic endpoints (CSS, RFS and intravesical recurrence-free), whereas SU was associated with superior renal functional outcome, showing a higher mean eGFR by 9.32 ml/min/1.73m² compared with RNU (p = 0.007) [42]. Consequently, postoperative renal function is generally well-preserved because of the sparing of nephron mass, while complications are manageable within experienced centers [37]. However, given the retrospective nature of the studies and no randomization, more studies (prospective and RCTs) are needed to backup these results.

Comparison with Endoscopic and Radical Approaches

Compared to endoscopic management, segmental ureterectomy allows improved local recurrence control, particularly in organ-confined distal ureter carcinoma. Studies have shown that distal ureterectomy is associated with significantly better local recurrence-free survival compared with endoscopic treatment [32]. Furthermore, adjustments for patient comorbidities reveal that kidney-sparing surgeries, including SU and TU, correlate with increased overall survival compared to nephroureterectomy, highlighting the functional benefits and potential survival advantages of nephron preservation [33]. These findings emphasize the necessity to integrate surgical kidney-sparing options into multidisciplinary care plans based on patient risk profiles.

Novel kidney-sparing therapies

UGN-101 (Mitomycin-Containing Reverse Thermal Gel)

Intracavitary chemotherapy is difficult in UTUC because of the rapid washout and short dwell time of the chemotherapeutic agent due to the physiology and anatomy of the region the tumor is located. UGN-101 is a novel formulation containing mitomycin C that uses a specific reverse thermal hydrogel that transitions from a liquid state at cold temperatures to a semi-solid gel at body temperature, in order to increase dwell time and consequently the efficacy of the treatment [43]. This gel gradually dissolves in 4-6 hours providing adequate exposure of the lesion to mitomycin, concurrently ensuring that systemic absorption is limited [44].

UGN-101 received FDA approval in April 2020 after the Olympus phase 3 trial demonstrated that 56% of the patients with low-grade UTUC remained in complete response after 12 months of induction therapy [45]. The treatment plan used consisted of six weekly instillations administered retrograde via ureteral catheter or antegrade through a percutaneous nephrostomy tube, while 50% of the patients with a complete response did not receive any maintenance instillations, and 59% received ≥1 maintenance instillation [45]. Regarding the long-term outcomes of UGN-101, the median duration of response from the patients who demonstrated a complete response was 47.8 months, while 75% of them had no evidence of recurrence in a median follow-up of 53.3 months [46]. A systematic review and meta-analysis of 20 studies involving 1,051 patients reported that complete response occurred in 49% of cases, with complications observed in 63% of patients, though the majority were of low grades [47]. Ureteric stenosis was the most common complication in both cases, occurring in 44% of patients in the OLYMPUS trial while in 35% of patients in the meta-analysis [46,47]. Preliminary evidence demonstrates that antegrade administration via nephrostomy tube may result in considerably lower rates of ureteral stricture (12%) compared to retrograde administration (32%), without compromising oncological efficacy [48]. Initial data also show that UGN-101 may also be used as adjuvant therapy following endoscopy nephron-sparing management with promising results [49,50].

Vascular-Targeted Photodynamic Therapy (VTP) with Padeliporfin

VTP with padeliporfin is a novel, nephron-sparing approach to treat UTUC, combining a photosensitizing agent, laser light, and oxygen in order to selectively destroy tumor tissue [51]. Padeliporfin, a water-soluble chlorophyll derivative that serves as the active photosensitizing agent, is injected intravenously, while an optical fibre, 20-40 mm diffuser, is positioned proximal to the tumor through the scope via retrograde upper tract endoscopy. Afterwards, the laser is activated for 10 minutes [52]. Results from the ongoing Phase 3 ENLIGHTED trial demonstrated a complete response rate of 73%, partial response rate of 13.5%, disease progression rate of 2.7% and an overall response rate of 86.5%, representing evidence of therapeutical potential [52]. The induction treatment consisted of 1-3 VTP treatments provided at four-week intervals until achieving a complete response or treatment failure. Patients achieving complete response proceeded to the maintenance treatment, where they were followed with endoscopic evaluation every three months, and VTP was provided for recurrent tumors in the period up to 12 months. The most frequent adverse events were predominantly Grade 1-2 and resolved within a few days [52].

Limitations and Future Prospects of Non-Surgical Kidney-Sparing Therapies

Even though these novel therapies appear as promising options, more prospective trials with longer follow-up intervals and larger sample sizes are needed to establish durable oncologic and functional outcomes. The integration of these therapies into clinical algorithms awaits further validation [45]. Perioperative chemotherapy and immunotherapy may complement these therapeutic options, as potential synergistic effects on disease control are under investigation [53].

Role of perioperative systemic therapies in kidney-sparing management

Chemotherapy Before and After Surgery

Systematic adjuvant chemotherapy in UTUC is used more in high-risk or invasive disease treated with RNU. Level 1 evidence supports that adjuvant chemotherapy after RNU improves oncologic outcomes [53]. Patients undergoing RNU, especially those with preoperative impaired renal function, may not be eligible for adjuvant chemotherapy due to the deterioration of renal function postoperatively. Better renal preservation observed in KSS may maintain a larger pool of patients who remain eligible for cisplatin-based adjuvant chemotherapy, compared to RNU [54]. However, literature contains no RCTs evaluating adjuvant chemotherapy after nephron-sparing approaches for UTUC. This represents a significant knowledge gap in the management of kidney-sparing approaches.

On the contrary, there is some evidence supporting better outcomes with neo-adjuvant chemotherapy followed by kidney-sparing approaches. In a prospective study of invasive UTUC treated with neoadjuvant platinum-based chemotherapy followed by organ-sparing approaches, there were no cases of progressive disease after four cycles [55]. Moreover, maximal tumor size decreased by 2.3 cm (mean maximal tumor size 4.17 cm before therapy), tumor complexity was reduced enabling organ-sparing approaches, better postoperative eGFR was observed compared to those undergoing RNU and two-year recurrence-free survival was better in the organ-sparing group (85% vs. 72%) [55]. Considering the aforementioned, neo-adjuvant chemotherapy may lead to an expansion of the indications for KSS to high-grade disease not feasible for RNU, downstaging the disease prior to surgery. However, more studies are needed to confirm the oncologic feasibility of this approach.

Immunotherapy and Novel Systemic Agents

Immunotherapeutic agents have been thoroughly investigated in recent years, with pembrolizumab, nivolumab, atezolizumab, durvalumab, and avelumab gaining FDA approval for UC [56]. However, those agents are not well studied for nephron-sparing management approaches of UTUC. The most comprehensive evidence for kidney-sparing approaches combined with novel systemic agents comes from a prospective pilot study integrating endoscopic thulium laser ablation (TLA) with perioperative disitamab vedotin (DV, an anti-HER2 antibody-drug conjugate) and checkpoint inhibitors (toripalimab or tislelizumab), demonstrating one-year local recurrence-free survival in 67% of the patients, two-year conversion-free survival (avoided salvage RNU) in 94% of the patients and OS in 94% of patients at two years with CSS 100% [57]. Moreover, molecular insights such as the aristolochic acid mutational signature suggest that certain UTUC subtypes may respond favorably to immune checkpoint inhibitors, opening avenues for integration with kidney-sparing strategies [22]. Trials evaluating perioperative immunotherapy combined with conservative surgeries may redefine the therapeutic landscape, emphasizing personalized, biology-driven care [53]. However, even as these kidney-sparing immunotherapy approaches show promise, they are not without limitations. Immune checkpoint inhibitors (ICIs) can provoke immune-related adverse events - for instance, colitis, pneumonitis, endocrinopathies, nephritis, and other organ-specific toxicities have been documented [58]. In clinical trials for urothelial carcinoma, approximately 10-20% of patients experience Grade ≥3 treatment-related toxicities on checkpoint inhibitors [59,60]. Likewise, antibody-drug conjugates such as disitamab vedotin carry chemotherapy-like side effects (e.g., peripheral neuropathy, cytopenias), with one real-world series reporting ~19.7% of patients having Grade 3-4 adverse events on a DV + ICI regimen [60]. These toxicities, while often manageable, mean that immunotherapy-based strategies may not be suitable for every UTUC patient, especially those with significant comorbidities or poor functional status. Therefore, immunotherapy is currently not well-studied yet in UTUC nephron-sparing management, and more prospective research is needed to evaluate its clinical efficacy.

## Conclusions

Kidney-sparing approaches in UTUC demonstrate promising results and safety profile concerning low-risk disease and carefully selected patients with high-risk disease. They seem to reduce morbidity while maintaining oncologic outcomes that are comparable to RNU when there is a rigorous follow-up plan. Consequently, management of UTUC has undergone a paradigm shift from RNU-centered approaches to nephron-sparing approaches. In the aforementioned carefully selected patients, endoscopic techniques and segmental ureterectomy can achieve oncologic outcomes comparable to the standard radical approach while preserving renal function. However, accurate preoperative risk stratification is crucial for ensuring better efficacy of the treatment selected. Clinical, imaging, and molecular risk stratification is essential for maximizing benefits while minimizing recurrence risk. Advanced imaging such as OCT and CLE and molecular biomarkers may enhance identification of the most suitable patients to be treated with KSS. Novel non-surgical modalities, including chemoablation with UGN-101 and photodynamic therapies, expand the scope of nephron preservation, offering less invasive treatment options with encouraging early results. Meanwhile, evolving data on neoadjuvant chemotherapy and immune checkpoint inhibitors highlight the potential synergy between systemic and kidney-sparing local treatments, suggesting future clinical trial directions.

On the contrary, significant research gaps exist, especially relating to long-term outcomes post-KSS, adjuvant therapies following conservative treatments, and integration of molecular biomarkers into clinical decision-making. More studies, particularly prospective studies and RCTs, are essential to address these areas in order to inform evidence-based guidelines. Enhanced diagnostic capabilities and evolving systemic therapies may help refine treatment paradigms, promoting personalized strategies that balance oncologic control with renal preservation.
